# A Plasmonic Nanoledge Array Sensor for Selective Detection of Cardiovascular Disease Biomarkers in Human Whole Blood

**DOI:** 10.1021/acsanm.4c02524

**Published:** 2024-08-16

**Authors:** Frank Tukur, Taylor Mabe, Mengxin Liu, Panesun Tukur, Jianjun Wei

**Affiliations:** † Department of Nanoscience, Joint School of Nanoscience and Nanoengineering, University of North Carolina at Greensboro, Greensboro, North Carolina 27401, United States; ‡ 3i Nanotech, Inc., 2901 E. Gate City Blvd, Greensboro, North Carolina 27401, United States

**Keywords:** surface plasmon resonance, biosensor, cardiovascular disease, biomarker troponin I, transmission spectrum

## Abstract

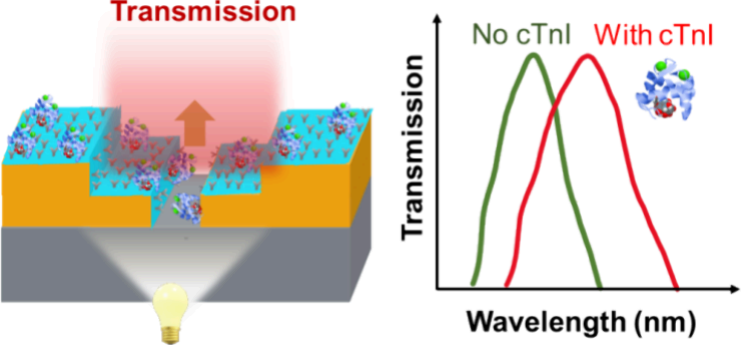

Optical sensors face challenges when detecting ultralow amounts of analytes in whole blood, including signal quenching due to optical absorption and false positives due to nonspecific binding. This study introduces gold nanoscale array features termed nanoledges (NLs), which interact with incident white light to produce a transmitted surface plasmon resonance (tSPR) signal. This extraordinary optical transmission (EOT) spectrum occurs in the near-infrared (NIR) region, thereby minimizing signal quenching caused by visible-light absorption from blood proteins and pigments. To develop a sensitive, selective, and label-free optical biosensor for detecting various levels of cardiac troponin I (cTnI) in very small volumes of whole blood samples, DNA aptamers are tethered to the NL surface, specifically binding to the cTnI biomarker. This biological binding activity alters the refractive index at the NL surface, causing a peak shift in the EOT spectrum and enabling quantification of cTnI levels. The NL array chip demonstrated high sensitivity for cTnI detection in buffer, human serum (HS), and human whole blood (HB), with detection limits of 0.079, 0.084, and 0.097 ng/mL, respectively. Control measurements using blank target mediums and those containing up to 125 ng/mL of other proteins, such as myoglobin, creatine kinase, and heparin, showed minimal interference and high specificity. The NL plasmonic array’s performance in biosensing underscores its promise for clinical analysis and its potential development as a point-of-care platform for early cardiovascular disease (CVD) diagnostics.

## Introduction

Recent advancements in biosensor technology have focused on a novel nanostructure-based “antenna″–aptamer–analyte sandwich approach.^1,2^ This signal transduction method involves three layers: the first layer is a functionalized “antenna” that captures the second layer, an aptamer, which in turn selectively captures the third layer, and the target analyte, producing measurable signals. The interaction at the interface generates an optical signal through the antenna, with the antenna’s properties determining whether the nanostructure acts as light scattering,^3^ fluorescence,^4^ surface-enhanced Raman scattering (SERS) colorimetric,^5^ and combined electrochemical and suface plasmon resonance (SPR)^6^ sensors. These systems typically use indirect signal transduction, converting chemical or biological responses into electrical or optical signals, offering the potential for developing integrated circuits and innovative biosensors.

A common feature in nanophotonic biosensors is the employment of thin metal films or nanoparticles coated or immobilized on glass, polycarbonate, or other metal substrates.^7^ The dominant limiting aspects with the use of thin-film metals is the requirement of a quartz prism to excite SPR.^8^ The complexity and bulkiness of these optical systems limits their versatility, miniaturization, and integration into point-of-care (POC) platforms.^9^ In the case of nanoparticle platforms, one encounters poor reproducibility due to the difficulty in synthesizing reproducible and uniformly sized particles. An alternative approach that eliminates the prism and offers high-precision reproducibility is the use of gratings in which the resonant condition is provided by diffraction of incident light. Grating systems have the ability to simultaneously excite localized surface plasmon resonance (LSPR) at the sharp edges of the grating nanostructure and surface plasmon polariton at the flat metal–dielectric interfaces to boost optical signal and sensitivity.^9^ Although nanogratings require more complex fabrication steps, it remains attractive due to its higher miniaturization and integration capabilities.^10^

Cardiovascular disease (CVD) continues to be the leading cause of death worldwide, and the importance of early detection and monitoring of CVD cannot be overstated.^11,12^ The presence of specific biomolecules, known as biomarkers, in the bloodstream plays a crucial role in predicting CVD. These biomarkers offer valuable insights into the physiological condition of the heart cells and exhibit detectable changes even in the early stages of the disease.^13^ One such biomarker, creatine kinase (CK), serves as an indicator for acute myocardial infarction (AMI) with exceptional specificity.^14^ However, its elevation in plasma following heart injury is relatively slow.^15,16^ Another widely used biomarker, cardiac troponin I (cTnI), is considered the gold standard for detecting myocardial tissue damage.^15−19^ Nonetheless, cTnI may not accurately diagnose a second heart attack that occurs 7 to 10 days after the initial one, mainly due to its short half-life (2 h) and associated features after the symptom onset, i.e., increasing in 2–3 h, peaking at 18–24 h, and remaining high up to 14 days.^20^ Hence, testing for multiple biomarkers can be beneficial for improving CVD risk prediction. For example, by utilizing both biomarkers such as cTnI in combination with myoglobin could improve the diagnostic accuracy for a second heart attack, even though myoglobin lacks specificity as a CVD marker and has a short half-life of 10–20 min.^21,22^

The CVD biomarker tests in whole blood enable fast CVD diagnosis while minimizing errors in sample pretreatments like centrifugation, dilution, separation, and transportation.^23^ A variety of detection methods including electrical or electrochemical method, colorimetry, chemiluminescence, fluorescence, enzyme-linked assays, SERS, and SPR have been reported for troponin analysis mostly in serum or plasma.^24^ However, analyzing whole blood samples using biosensors is challenging due to issues like elevated background levels, baseline fluctuations, and sensitivity changes caused by nonspecific binding of interferents. One strategy to reduce these effects is sample filtering, but it adds complexity and cost.^25^ While optical biosensors have been explored for direct analysis in complex mediums,^26,27^ they struggle with problems of light scattering and absorption by optically active biological species like hemoglobin, thus limiting sensitivity and accuracy by hindering the detection of analytes at low concentrations.^28^ A modern approach based on plasmonic nanomaterials amplifies readout signal and enhances sensitivity for low-concentration analytes in complex mediums.^29,30^ Notably, recent technologies that are based on the sandwich nanostructure have found relevance in analyzing biomarkers for disease diagnosis and prognosis.^31,32^ While the pattern of the detection procedure differs for these advanced biosensors, a common feature is the employment of thin metal films or nanoparticles coated or immobilized on glass, polycarbonate, or other metal substrates.^7,33^ These components present an ideal platform for the development of sensitive diagnostic devices with operational simplicity.^8^ The key limitation shared by most optical sensors includes the dependence of the signal intensity on the sensing samples and environment. For example, the use of human whole blood samples may cause partially quenched signal transduction due to light absorption compared to a clearer medium such as the saliva, sweat, urine, or serum.^28^ To date, chip-based plasmonic devices with well-controlled, reproducible nanostructures in whole blood detection of troponin were less reported compared to nanoparticle-based sensors.^2^

**Figure 1:**
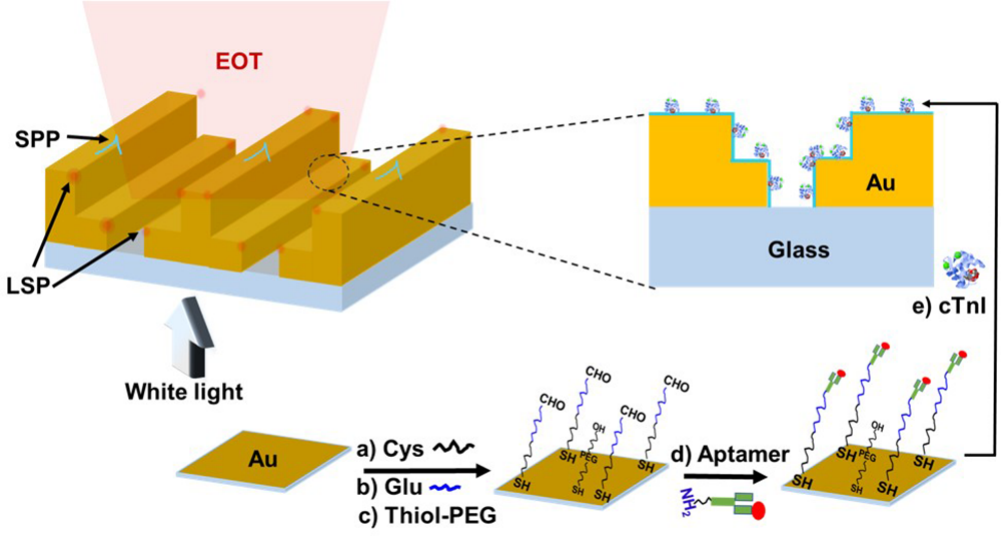
Schematic illustration of the sensing principle and the surface preparation for selectively binding the protein biomarker cTnl at the NL gold surface, showing sequential steps for (a) surface functionalization with the monolayer of cystamine (Cys), (b) tethering of the glutaraldehyde (Glu) cross-linker, (c) capping of the exposed gold surface using thiol PEG, and then (d) aptamer attachment to the Glu terminal via imine bond for (e) detection of cTnI by the specific-binding aptamer.

In this report, we present a portable (25.4 × 12.2 mm) label-free plasmonic device for detecting cTnI as a model protein biomarker in human whole blood. The sensing principle shown in Figure 1 is based on the EOT signal changes through the NL array with the biological binding event. The light transmission through a subwavelength aperture in metal films is aided by surface plasmon excitation, which mediates light tunneling and transmission, hence giving birth to the phenomenon of EOT.^29^ The wavelength of optical transmission through the NL aperture can be approximated by eq 1.^34^
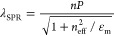
1

From eq 1, the spectral characteristics of the EOT depend on the effective refractive index (*n*_eff_) at the metal/dielectric interface; the height (*h*), width (*W*), and periodicity (*P*) of the nanoaperture; and the resonance wavelength (λ) at the phase matching condition. Since the size, shape, and geometry of the metal aperture determine the efficiency of surface plasmon polariton (SPP) excitation, the SPR-mediated EOT is also dependent on the geometry of the nanoaperture. The wavelength sensitivity of EOT to changes in RI at the metal surface informed our decision to use the NL EOT-based system to interrogate biological binding interactions at the NL surface.

Additionally, we demonstrate that the optical transmission signal of an NL array antenna in the near-infrared (NIR) regime and its sensitivity to changes in the near-surface refractive index have been exploited in optical biosensing to optimize signal readout in whole blood samples. The active plasmonic NL array as the “antenna” is firstly functionalized with cystamine/glutaraldehyde. Then, DNA aptamers integrated onto the nanoledge (NL) array enable the selective recognition and binding of cTnI without additional sample preparation or purification steps. The biosensor overcomes limitations associated with commercial methods, e.g., enzyme-linked immunosorbent assay (ELISA), chemiluminescence enzyme immunoassay, and immunofluorescent labeling, which use antibodies or cellular membrane receptors for cTnI detection. Antibodies have low stability, resulting in short shelf life and susceptibility to denaturation.^35^ Additionally, antibodies are costly and cannot be synthetically produced.^36,37^ These conventional methods often have low sensitivity, have long processing times, and require large blood samples, performing best in transparent test fluids rather than whole blood or serum unless diluted significantly.^38,39^ Notably, the synthetic DNA aptamers as recognition agents are more robust for selectively recognizing cTnI, overcoming these drawbacks.^40−42^

In our research, we successfully demonstrated that employing the synthetic DNA aptamer-modified NL system mitigates these challenges. Notably, we achieved a detection time of just 20 min, requiring a sample size of less than 10 μL. Furthermore, our system exhibits sensitivity to cTnI even in undiluted blood samples. The sensing scheme utilizes EOT in the NIR range, minimizing interference from absorption and/or fluorescence background in biological samples. The binding of cTnI to the functionalized NL arrays alters the surface refractive index, which can be monitored by measuring shifts in the EOT wavelengths. In brief, this work represents significant advances in reproducible NL device production and stable and reusable surface functionalization for aptamer–cTnI binding reaction, which tackles the problems in whole blood and realizes sensitive and reliable cTnI detection. More importantly, while only the sensing of cTnI in whole blood is reported, the biosensor technology can be potentially designed for a multiplexed platform rendering high-throughput analysis of a multimarker protein panel for CVD diagnosis and prediction.

## Results and Discussion

### NL Array Fabrication and Structure

**Figure 2:**
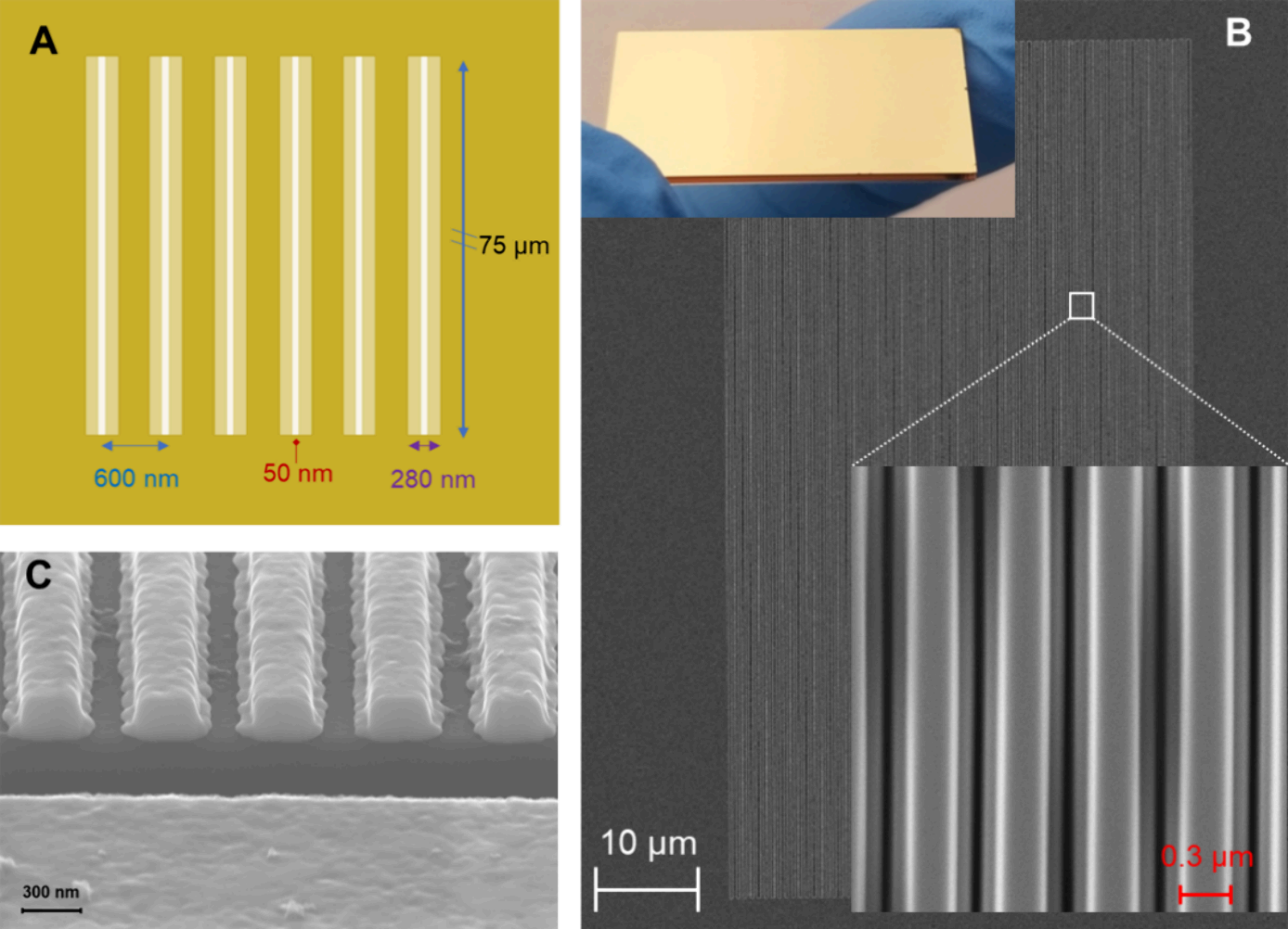
(A) Top-down view of the design of the NL array. (B) SEM image of the final fabricated NL array with inserted photo of the gold slide. (C) High-resolution SEM image for a close cross-view of the NL structure.

The NL array was fabricated on a 250 nm-thick gold film, supported on a transparent glass substrate using an electron beam direct patterning method. Each chip device, measuring 25.4 × 12.2 mm, consisted of four NL arrays and one reference burnt box without a gold coating. Notably, the four NL arrays on the chip were identical and were used for reproducible studies. The NL arrays and reference burnt box were designed to be approximately 45 μm × 75 μm in dimension and were spaced 1 mm apart (Figure 2A) to prevent interference from transmitted light via neighboring NL arrays. Figure 2B displays the scanning electron microscopy (SEM) image of the NL array. Figure 2C provides a high-resolution SEM image of the cross-section view of the NL array structure, which shows the roughness at the nanoscale level.

### Sensitivity of EOT to Refractive Index Changes

Methanol, water, ethanol, and isopropyl alcohol solvents were used to determine the bulk RI sensitivity of the gold NL-SPR sensor. The solvents were selected due to their small differences in RI. This makes it possible to determine if the device would be responsive enough to accurately determine small wavelength shifts when the solvents access the NL surfaces. To overcome the hydrophobic effect of the bare gold surface and enhance the access of the solvent or analyte to the sensing area, the chip was subjected to surface pretreatment. Traditionally, exposing the gold surface to Ar/water plasma or Ar plasma increases wettability. However, this approach increases the metal surface roughness and dampens the evanescence field. For this reason, we carried out surface pretreatment by irradiating the chips with UV/ozone. During the process of UV/ozone treatment, generated oxygen radicals react with water molecules present in air to form hydroxide radicals. These short-lived, highly reactive species can react with bonds on the surface of the substrate, resulting in the formation of high-energy hydroxide groups, making the surface more hydrophilic and increasing the surface wettability.^43,44^ This approach enhances wettability without compromising the integrity of the metal surface.^43^ Hence, the evanescent field is expected to remain unaffected.

Figure S1 shows the EOT spectra of the solvents after the NL structure. The primary EOT peaks red-shifted with a linear dependence on RI. From the linear plot, the sensitivity is found to be 384.1 ± 4.6 nm/RIU. The increase in sensitivity from ∼118 (NL before UV/ozone treatment) to ∼384 nm/RIU (after UV/ozone treatment) may be attributed to the enhancement in surface wettability and accessibility to the NL surfaces.^43^ Since the solvents have minimal to no chemical tendency to bind or react with the treated Au surface, the observed wavelength responses are taken to come from changes in the RI of the bulk medium rather than surface binding.

Surface binding was achieved through chemical functionalization of NL surfaces with cystamine/glutaraldehyde, as depicted in Figure 1. This step served the purpose of enhancing surface wettability and acting as a molecular linker for the DNA aptamer. Figure S2 shows the EOT spectra measured at three stages of the characterization process: bare gold NL array substrate, after the cystamine/glutaraldehyde/PEG self-assembled monolayer, and after aptamer immobilization. The red shift in the EOT peak indicated molecular binding occurrence at each step. Subsequently, the binding kinetics of the cTnI target to the aptamer was monitored, revealing a further red shift in EOT peak wavelength position as incubation time increased, reaching equilibrium after 20 min (Figure S3). An incubation time of 20 min was adopted for subsequent cTnI detection experiments. To establish a baseline for the biosensor and account for any nonspecific interactions due to solvent interference or noise signals from the analytical instrument, a blank measurement without cTnI was initially taken.

### Cardiac Biomarker (cTnI) Detection

**Figure 3:**
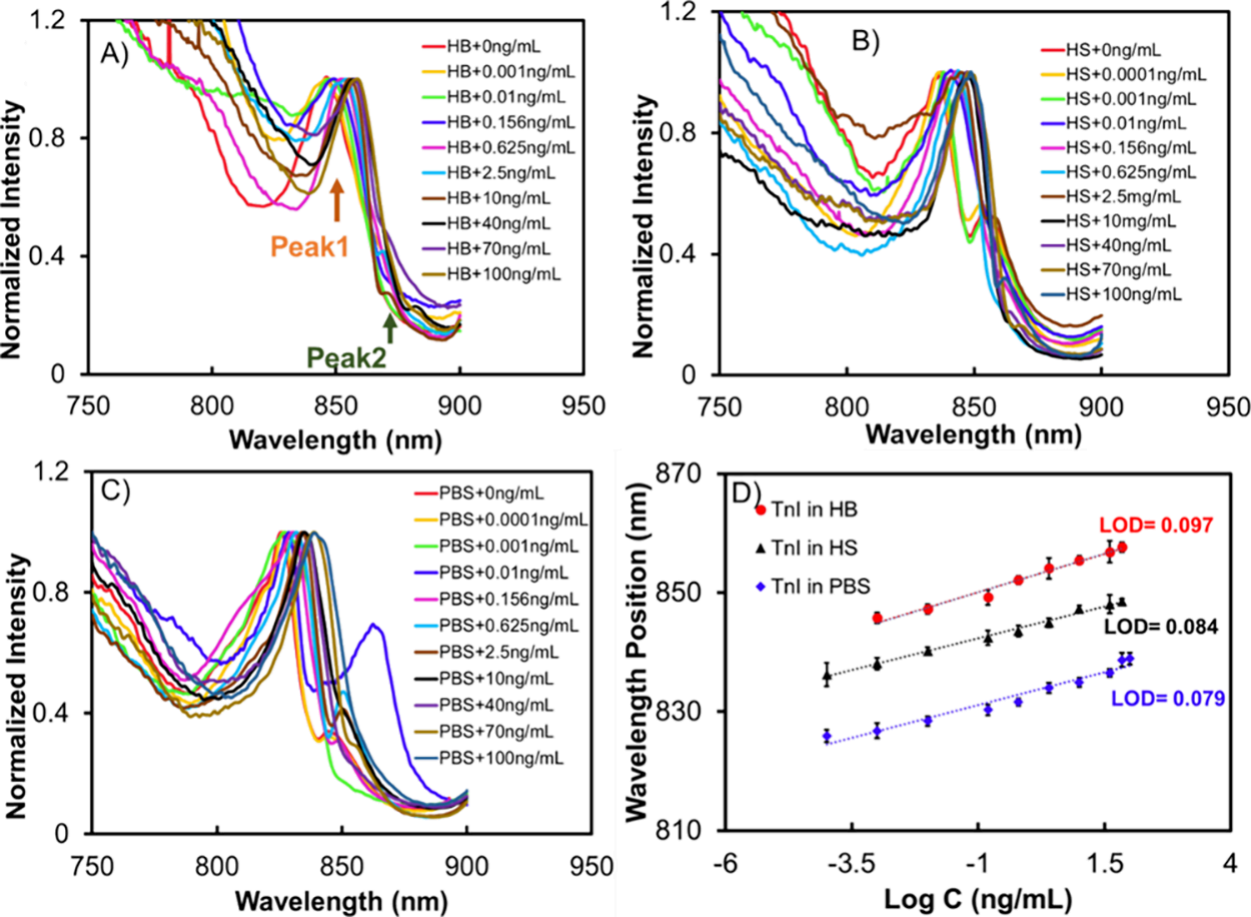
EOT peak curve plots recorded upon TnI incubation with aptasensor at different concentrations (0.0001, 0.001, 0.01, 0.156, 0.625, 2.5, 10, 40, 70, and 100 ng/mL) in (A) human whole blood, (B) human serum, and (C) PBS. (D) Calibration plots for cTnI in PBS (black), human serum (blue), and human whole blood (red). Figure S6 shows representative plots of raw data plots with smoothed plots. Figure S7 shows the enlarged spectral shifts for cTnI measurements at different concentrations in the three mediums.

The performance of the NL sensor in detecting cTnI was assessed at various concentrations of cTnI (0.0001, 0.001, 0.01, 0.156, 0.625, 2.5, 10, 40, 70, and 100 ng/mL) spiked in HB (Figure 3A). In the low-concentration range of cTnI (0.0001 to 0.001 ng/mL), we did not observe a significant shift in the wavelength position of the EOT relative to the background signal (sample without cTnI). However, as the concentration of cTnI increased within the higher range (0.001 to 70 ng/mL), we observed a significant red shift in the wavelength, which correlated with the concentration increase. This shift indicated evidence of an aptamer-cTnI binding reaction. To determine the limit of detection (LOD) of the biosensor, we employed the formula LOD = 3σ/*S*, where σ represents the standard deviation of the blank signal (0.05) and *S* denotes the slope of the calibration plot.^45^ By applying this formula, we determined the LOD to be 0.097 ng/mL. Additionally, the linear range of detection spanned from 0.001 to 70 ng/mL. The remarkably low LOD achieved can be attributed to the NL array, which generates an enhanced near-infrared (IR) field through the excitation of cavity-coupled LSPR and SPPs, as indicated by the presence of peaks 1 and 2 (Figure 3).^46,47^ The resonance location of the LSPR is determined by the width of the NL aperture.^34,48^ Apart from serving as an optical signal booster, the resonance wavelength of the NL antenna in the NIR range enhances the signal readout by minimizing absorption interference, which predominantly occurs in the 540–576 nm range for major blood components such as hemoglobin.^49^

At the same time, we conducted evaluations on the spiked concentrations of cTnI in HS and PBS for a comparison. As the concentration of cTnI increased, we observed red shifts in the position of the EOT peak wavelength, corresponding to the changes in the surface refractive indexes (Figure 3B,C). In Figure 3D, the calibration plot for cTnI in HS (represented by the black dotted line) showed the LOD to be 0.084 ng/mL and the linear detection range spanned from 0.0001 to 70 ng/mL. Similarly, the analysis of the LOD in PBS samples gave 0.079 ng/mL LOD and the linear range of detection extended from 0.0001 to 70 ng/mL.

Significantly, the linear calibration lines depicted in Figure 3D for cTnI in the three different media (PBS, HS, and HB) did not overlap. Instead, they exhibited a noticeable offset toward higher wavelengths as the analyte medium transitioned from PBS to HS and HB, respectively. This linear offset can be attributed to variations in the refractive index of PBS, HS, and HB. This highlights the NL sensor’s response to changes in the bulk refractive index and the sensitivity to the binding events at the surfaces.

The binding stage analysis indicates that each interaction, starting from surface functionalization to cTnI detection, induces modifications in the refractive index at the metal surface due to the film thickness changes. This effect becomes evident through the observed spectral shift, where the wavelength position changes from approximately 800 nm (nonfunctionalized surface) to 840 nm (cTnI in PBS), 849 nm (cTnI in HS), and 859 nm (cTnI in HB) (see Figures S4 and S5 for binding event analysis). This shift in the resonant wavelength position serves as a clear indication of the sensing capability of the device. A more detailed analysis of the binding events at the NL surfaces by considering the effective thickness changes, and thus the surface refractive index change, is provided in the Supporting Information. The analysis presents a more quantitative understanding of the approach for surface functionalization and cTnI binding to the aptamer by the layer thickness changes and corresponding RI change.^50^

To this end, the linear detection range and the LOD values obtained for cTnI in PBS, HS, and HB demonstrate the NL sensor’s potential to differentiate between healthy individuals and afflicted patients. Normal cTnI levels typically fall within the range of 0–0.04 ng/mL, while levels above 0.40 ng/mL are indicative of AMI.^2,51^ Hence, it suggests that the NL sensing performance could potentially allow for identification of AMI cases and distinguish them from individuals with normal cardiac biomarker levels. Currently, we are working to develop a prototype device for real clinical sample applications in the next stage.

To test the device’s response to nontarget species and assess its specificity, we introduced known amounts of interferents, namely, myoglobin, CK, and heparin, into PBS buffer at concentrations ranging from 0.156 to 125 ng/mL. This approach aimed to mimic the multicomponent nature of human blood at elevated levels and evaluate the sensor’s ability to discriminate against these interfering substances. The concentration range was selected to account for the variation in the concentrations of the chosen interferents in real samples. We specifically selected myoglobin (6–85 ng/mL in blood) and CK (1–4 ng/mL in blood), as they are also released into the bloodstream during cardiac malfunction.^52,53^ Furthermore, heparin, a commonly used treatment for heart disease, was included to ensure that its presence does not interfere with the device’s ability to accurately monitor cTnI levels during treatment.^18^

**Figure 4:**
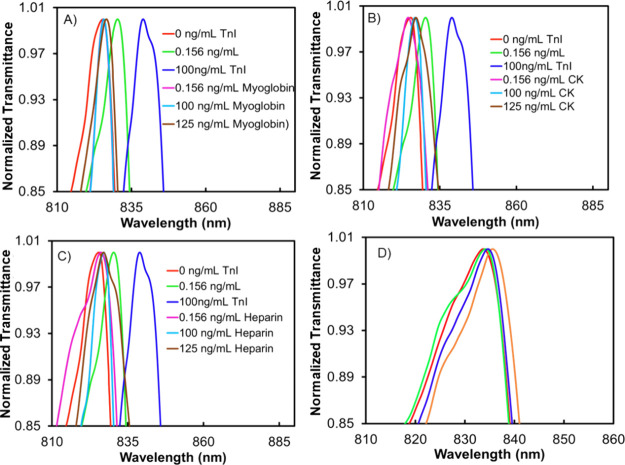
Normalized EOT spectra of the NL sensor chip with (A) myoglobin, (B) heparin, and (C) creatin kinase in PBS, and (D) EOT spectra recorded using four different fabricated aptasensors showing the reproducibility of the aptasensor at 2.5 ng/mL cTnI in PBS. The full spectrum for Figure 4A–D is presented in Figure S8.

To minimize nonspecific fouling, we attached an amine-modified cTnI aptamer to the sensor active area using cystamine and glutaraldehyde as cross-linkers. In addition, to ensure comprehensive coverage, any unoccupied regions on the gold surface were effectively capped with thiol-poly(ethylene glycol) (PEG) while the exposed carbonyl groups on glutaraldehyde were efficiently capped and deactivated with glycine. Despite the various doses of myoglobin, CK, and heparin, only minimal fluctuations in the EOT wavelength position were observed at 125 ng/mL of interferents compared to the significant shifts observed for cTnI at a lower concentration of 0.156 ng/mL (Figure 4). The response at 100 ng/mL cTnI exceeds those of interferents by at least 12 times. This observation indicates the poor binding of the interfering species to the sensing area functionalized with cTnI-specific aptamers, demonstrating the specificity of the aptamer’s binding to cTnI. It was reported that the presence of blood platelets, potentially leading to physical clogging at the sensor surface, might introduce variations in the estimated thickness,^54^ which was not observed in this study. We believe, other than the PET treatment, that the unique NL geometry and the dimensions may block the access of blood platelets (2 μm size) into the NL channels. In summary, the experiments involving interferents and their negligible impact on the EOT wavelength shift further support the device’s ability to selectively detect cTnI.

In order to investigate the reproducibility of the cTnI aptasensor, the sensor-to-sensor variation of four differently fabricated devices was determined using 2.5 ng/mL cTnI in human serum. The EOT responses from each sensor device were recorded separately. As shown in Figure 4D, the wavelength positions for all four devices differ by about ±0.1% relative standard deviation. This confirms the reproducibility of the sensor device.

### Reproducibility, Stability, and Reusability of the NL Aptasensor

It is desirable for an aptasensor to be stable, a property that will allow it to be stored and deployed easily to the location of application without losing its ability for precise analyte detection.^45^ To assess the stability, seven identical aptamer-immobilized NL devices were stored in a refrigerator at 4 °C and measured at a 3-day interval before and after incubating with 10 ng/mL cTnI in PBS at room temperature. Figure S9 indicates that the transmitted wavelength position on the first 3 days is consistent with the one observed after 21 days. The sensors maintained above 99 ± 0.8% of the original measurement signal after 21 days. With an economic implication in mind, we further investigated the reusability of the aptasensor. Owing to the strong affinity between the −SH groups of cystamine and the gold NL surface, coupled with the durable imine bond (bond energy of 644 kJ/mol) formed between the carbonyl and amine moieties of glutaraldehyde and the aptamer, the interaction between the aptamer and cTnI is facilitated by hydrogen bonds, van der Waals forces, and electrostatic interactions (with bond energies <569 kJ/mol).^55^ This provides a relatively weaker link to aptamer, allowing the aptamer layer to be regenerated after binding with the cTnI protein.^55,56^ This was achieved by inserting the used device after binding cTnI in a 95 °C deionized water for 5 min.^57^ Notably, Au–S bonds formed by using thiol compounds like cyclic disulfide are thermally unstable especially at higher temperatures.^58^ However, monolayers formed with thiol or acyclic disulfide exhibit stability and packing density that tend to improve at higher temperatures above 80 °C than at temperatures below 40 °C.^59^ In this report, cystamine, an acyclic disulfide, was used for gold surface functionalization. Figure S10 shows the recovery of the aptamer layer after multiple regeneration cycles. A noticeable loss of aptamer regeneration peak signal was observed in the fourth regeneration cycle likely due to thiol monolayer distortion as well as feeble affinity of cTnI to the aptamer whose stability is impacted by the intermittent heating and cooling cycles.^45^ However, 99.5% signal recovery was obtained after three regeneration cycles, which demonstrate the superior reusability of the NL aptasensor.

### Comparison of cTnI Detection

In comparison to other sensing assays (Table 1), our methodology demonstrates a competitive turnaround time and sensitive signal response to low cTnI concentrations in different mediums. For the direct detection of cTnI in whole blood, our developed device exhibits an LOD lower than those of existing methods, such as the reported SPR/electrochemical sensor, by 0.003 ng/mL.^2^ Furthermore, we achieved a linear detection range exceeding that of previously reported data for cTnI detection in whole blood by over two to three orders (refer to Table 1 for comparison). While certain biosensors have demonstrated lower LODs, it is noteworthy that they were primarily tested in either HS or PBS medium. These sensors were not specifically designed for whole blood sensing, likely due to their susceptibility to interferences. In contrast, the NL device is engineered to mitigate such challenges, making a significant improvement for the quantitation of cTnI in human whole blood. It could greatly complement the EKG monitor used by health emergency workers and be superior for more direct, early detection of the risk of heart attack. This capability further promotes the use of our technique to further develop POC devices for more disease diagnostics by detecting protein biomarkers in whole blood samples.

**Table 1:** Detection Methods and Performance of Different cTnI Assays

**detection method**	**transducer**	**target**	**detection range**	**LOD**	**real matrix**	**time (min)**
SPR/fluorescence^60^	Au chip	cTnI	84 aM–350 pM	3.5 × 10^–7^ ng/mL	PBS	60
electrochemical^1^	Au electrode	cTnT	0.05–5 mg/mL	17,000 ng/mL	serum	30
chemiluminescence^61^		cTnI	2–25 μg/L	600 ng/mL	PBS	10
				60,000 ng/mL	plasma	
				70,000 ng/mL	serum	
electrochemical^51^	carbon nanofiber	cTnI	0–1 μg/mL	0.2 ng/mL	PBS	60
SPR/electrochemical^2^	electrode	cTnI	0.015 ng/mL	0.015 ng/mL	PBS	30
			0.1 ng/mL	0.1 ng/mL	whole blood	
SPR^62^	Au stripes	cTnI	1–1000 ng/mL	0.028 ng/mL	PBS	40
SPR^63^	Au/polydopamine	cTnI		1.25 ng/mL	HS	40
SPR^64^	Au film	cTnI	0–160 ng/mL	0.068 ng/mL	PBS	5
SPR^65^	Au nanorod	cTnT	7.6 fg/mL–910 μg/mL	8.4 × 10^–6^ ng/mL	PBS	10s
dielectric^66^	silica fiber	cTnI	0.1–10 ng/mL	0.03 ng/mL	Tris–urea buffer	60
dielectric^67^	Si grating	cTnI	0.1 ng/mL–80 μg/mL	0.1 ng/mL	HS	4
**this study**	Au chip	cTnI	0.0001–70 ng/mL	0.079 ng/mL	PBS	20
			0.0001–100 ng/mL	0.084 ng/mL	serum	
			0.001–100 ng/mL	0.097 ng/mL	blood	

## Conclusions

In brief, the novelty of the subwavelength NL system lies in its ability to interact with incident light and excite localized and propagating surface plasmons without the need for a prism coupler, resulting in EOT phenomena that are applied effectively in biosensing. By fabricating the NL channels on a metallic gold surface at inner and outer ledges of widths 50 and 280 nm, respectively, the device exhibits NIR range EOT signals that are highly sensitive and rapid responsive (in minutes) to the surface RI changes caused by aptamer-protein binding events at the NL surfaces. This work reports on high sensitivity for the detection of cTnI over a wide range of concentrations up to 100 ng/mL with excellent detection limits of 0.079, 0.084, and 0.097 ng/mL in different mediums, namely, PBS, human serum, and notably, human whole blood, respectively. Remarkable selectivity against interferents, such as spiked myoglobin, creatin kinase, heparin, and the present species in whole blood, was achieved by functionalizing the sensor with highly cTnI-specific aptamers. The advantage of the NIR EOT signal was realized by alleviating the background noise of biological species in the blood. Considering the stability, reusability, and high sensitivity toward cTnI in different biological mediums, this chip-based plasmonic technology could potentially pave a revenue to the development of a POC analytical tool for multiple cardiac protein biomarker analysis in preclinical or clinical applications.

## Materials and Methods

### Materials and Reagents

Cystamine dihydrochloride (98%), glutaraldehyde solution, thiol PEG (SH-PEG), glycine, human serum (male; blood type, AB, cat. no. H4522), and heparin sodium salt were all purchased from Sigma-Aldrich and used without further purification. Creatine kinase (cat. no. 9076-ck), myoglobin (cat. no. NBPI-50959), human heart troponin I (cat. no. 648480), and phosphate-buffered saline (PBS) were obtained from Fisher Scientific. The cTnI-specific aptamer 5′-/5AmMC6/-CGT GCA GTA CGC CAA CCT TTC TCA TGC GCT GCC CCT CTT A-3′ (cat. no. 316487191) was ordered from integrated DNA Technologies. Aptamer selection was based on the reported high-selectivity performance toward cTnI.^68,69^ Methanol, ethanol, and isopropanol were purchased from Sigma-Aldrich and used without further purification.

### Optical Characterization

The optical setup shown in Figure 1 was used to acquire the EOT characteristic of the device. The microscope used was connected to an external tungsten halogen white light source (LS-1, Ocean Optics Inc., USA) via an optical fiber. The white light is launched on to the Au NL by passing through the microscope’s condenser. The characteristic transmitted light spectra are then measured by the spectrometer, which is connected to a computer for data acquisition and processing.

To get the EOT spectral response that is specific to Au NL structures, a reference spectrum was obtained from a burnt box (with no NL structure) and a dark spectrum was acquired from a region of the chip with no NL or burnt box but a 250 nm-thick Au film. The measured transmission spectra of the nanoslit sensor probe was thus obtained by eq 2.^50^
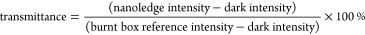
2

### Functionalization and Aptamer Immobilization

The immobilization of the cTnI aptamer at the plasmonic sensor surface is similar to a procedure previously reported.^70^ First, the chip was subjected to thorough cleaning starting with ethanol rinse, followed by O_2_ plasma cleaning (5 min, 100 W, 180 mTorr O_2_ pressure, −783 V DC bias; South Bay Technology PC-2000 Plasma Cleaner), and then ethanol rinse, N_2_ drying, and UV/ozone treatment using a Bioforce UV/Ozone ProCleaner. Then, a self-assembled monolayer (SAM) was formed by inserting the chip in a 25 mM solution of cystamine (containing 50% ethanol to enhance pact SAM on the Au chip) followed by a catalytic irradiation in a microwave synthesizer (50 W, 50 °C) for 5 min. As a result, the two disulfide bonds within cystamine break to form two sulfur–Au bonds at the surface of the chip.^70^ After irradiation, the chip was rinsed with 90% ethanol and then with DI water. Next, the chip was placed in a 2.5% glutaraldehyde solution and irradiated again (50 W, 50 °C, 5 min) to facilitate the cross-link between amine functional groups of cystamine with the carbonyl functional groups in glutaraldehyde to form an imine bond. This leaves a terminal aldehyde group, to which an amine-modified aptamer can bind. The gold surface area that remains uncovered by the cystamine SAM was further capped by irradiating (50 W, 50 °C) the chips in a solution containing 20 μM SH-PEG 500 for 5 min and washed thrice with DI water and N_2_ dried. The sensors were then subjected to aptamer immobilization by drop casting 60 μL of solution of the aptamer onto the sensing area and incubated at room temperature overnight. During this time, a wet Kimwipes in a covered Petri dish was used to maintain chip humidity. After DI water rinsing and N_2_ drying, the chip was further incubated in a 0.2 M solution of glycine for 5 min to block unprotected carbonyl (C=O) groups on glutaraldehyde and washed with DI water, N_2_ dried, and stored at 4 °C until use.

### Detection of the cTnI Biomarker

After the immobilization of the aptamer, the NL-SPR sensor is ready for detection of cTnI. A stock solution of 100 μg/mL of cTnI solution was spiked in a buffer, human serum, and human whole blood to yield a working solution of different required concentrations. In order to probe the binding of cTnI to the aptamer-modified surface, the EOT method was carried out. First, the sensors were incubated in mediums containing a cTnI sample of concentrations in the range 0.0001–100 ng/mL for 20 min. Then, by analyzing the peak shift of EOT at varying concentrations of cTnI in both biological and nonbiological mediums, the local refractive index changes due to cTnI–aptamer interaction were obtained. Note that for each medium used, a reference EOT was obtained without cTnI. To determine the specificity of the aptasensor, a similar approach to the one described above was used to develop a control experiment and obtained signals resulting from the presence of myoglobin, CK, and heparin (0.156–125 ng/mL) interferents in human serum. Myoglobin and CK were selected for this study because they are also myocardial biomarkers similar to cTnI.^15^ Heparin is a common treatment for blood cloth, and it was selected to determine if such a treatment could interfere with the sensor’s activity.

## Supporting Information Available

EOT due to solvents at different refractive indexes for sensitivity evaluation; EOT due to SAM formations and aptamer immobilization; EOT recorded upon cTnI incubation with aptasensor at different binding times; graphical fit of concentration-dependent wavelength peak position; fependence of adlayer film thickness on the change in wavelength position and cTnI concentration; representative plots of raw and smoothed EOT spectral curves; EOT spectra showing selectivity and reproducibility of NL sensors; stability of aptasensor measured EOT spectra at a 3-day interval for 21 days; reusability of aptasensor after four regeneration cycles; and adlayer thickness as a function of concentration and wavelength peak shift (PDF)
