# Supplementary material for: A Plasmonic Nanoledge Array Sensor for Selective Detection of Cardiovascular Disease Biomarkers in Human Whole Blood
Source: ACS Appl Nano Mater. 2024 Aug 16;7(17):20024–33. doi: 10.1021/acsanm.4c02524 (PMC11406491; doi:10.1021/acsanm.4c02524)
Supplement: Supplementary file 1 — an4c02524_si_001.pdf [file an4c02524_si_001.pdf]

Supporting Information

A Plasmonic Nanoledge Array Sensor for Selective Detection of Cardiovascular Disease Biomarker in Human Whole Blood

Frank Tukur,[†] Taylor Mabe,[‡] Mengxin Liu,[†] Panesun Tukur,[†] Jianjun Wei[†‡]*

[†] *Department of Nanoscience, Joint School of Nanoscience and Nanoengineering, University of North Carolina at Greensboro, Greensboro, NC 27401, USA.*

[‡]*3iNanotech, Inc, Greensboro, NC, USA*

*Corresponding Author: j_wei@uncg.edu

Table of Content

Fig. S1. EOT due to solvents at different refractive indexes for sensitivity evalluation.

Fig. S2. EOT due to SAM formations and aptamer immobilization.

Fig. S3. EOT recorded upon cTnI incubation with aptasensor at different binding times.

Fig. S4. Graphical fit of concentration dependent wavelength peak position.

Fig. S5. Dependence of adlayer film thickness on the change in wavelength position and cTnI concentration.

Fig. S6. Representative plots of Raw and smoothed EOT spectral curves.

Fig. S7. Enlarged EOT peak spectra for cTnI measurement in (A) Whole blood sample (B) Human serum (C) PBS buffer.

Fig. S8. EOT spectra showing selectivity and reproducibility of NL sensors.

Fig. S9. Stability of Aptasensor measured EOT spectra at three days interval for twenty-one days.

Fig. S10. Reusability of Aptasensor after four regeneration cycles.

Table S1. Adlayer thickness as a function of concentration and wavelength peak shift.

The principle of the sensing scheme is based on the extraordinary optical transmission (EOT) of light through the NL array. According to Bethe's theory, the diffraction limit of light restrict light to pass through an orifice smaller than its wavelength.[1] However, subwavelength apertures in metal films can transmit light due to surface plasmons excitation which mediates light tunnelling and transmission, and hence giving birth to the phenomenon of EOT.[2,3] The wavelength of optical transmission through the NL aperture can be approximated by Eq. 1.[4-5]

$$\lambda_{SPR} = \frac{nP}{\sqrt{1 + n_{eff}^2 / \varepsilon_m}} \qquad (1)$$

From Eq. 1, the spectral characteristics of the EOT depends on the effective refractive index (n_{eff}) at the metal/dielectric interface, the hight (h), width (W) and periodicity (P) of the nanoaperture and the resonance wavelength (λ) at the phase matching condition. Since the size, shape and geometry of the metal aperture determines the efficiency of SPP excitation, the SPR mediated EOT is also dependent on the geometry of the nanoaperture. The wavelength sensitivity of EOT to changes in RI at the metal surface is what informed our decision to use the NL EOT-based system to interrogate biological binding interactions at the metals surface.

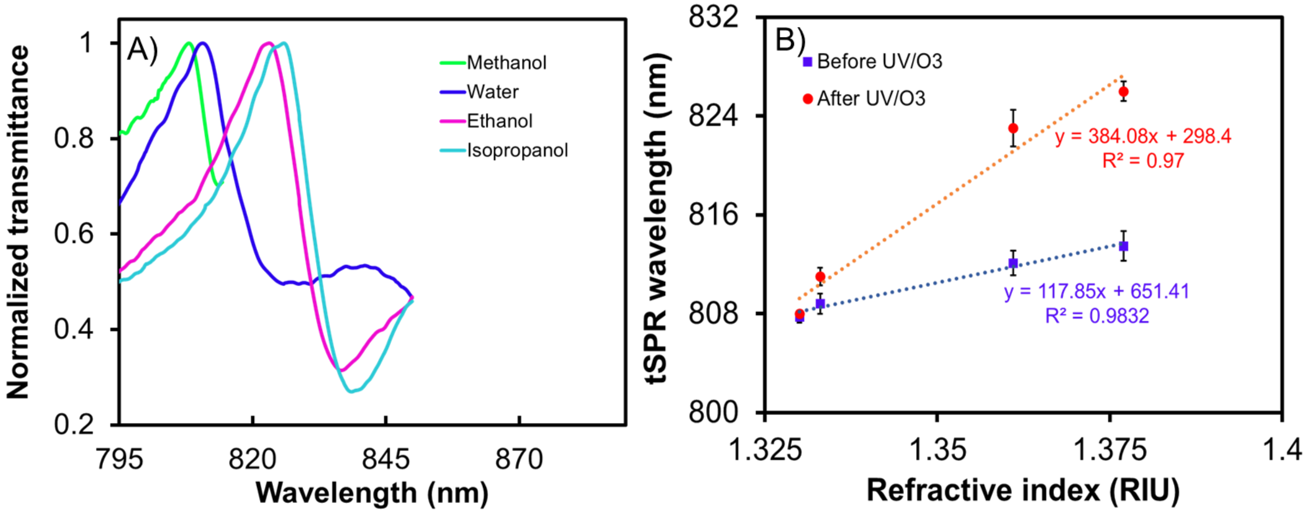

Figure S1. (A) measured transmittance (EOT) spectra for the NL-SPR probe in various solvents. (B) Linear fit of the EOT peak wavelength.

Note that the inner ledges are designed to enable SPR excitation for EOT and to capture the biomarkers and thus provide increased sensitivity to the device.[6] Initially, clean gold nanoledge is chemically modified to have a terminal carbonyl group that covalently bind with an amine modified cTnI aptamer.[7] Since the aptamer is designed to specifically target cTnI protein, incubating the aptamer modified sensor with cTnI results in another binding interaction.

Following the detection of different concentrations of cTnI in whole blood, human serum, and PBS buffer, we established a linear regression equation, given by $\lambda(\mathrm{nm}) = 2.64 \pm 0.085 \text{ x} \log \mathrm{C} \left(\frac{\mathrm{ng}}{\mathrm{mL}}\right) + 852.75 \text{ (nm) with } R^2 = 0.982$, λ(nm) = 2.14±0.09 x log C (ng/mL) + 844.47 (nm), with R^2 value of 0.993, and λ(nm) = 2.2±0.16 x log C (ng/mL) + 833.3 (nm), with R^2= 0.980, respectively (**Figure 3**).

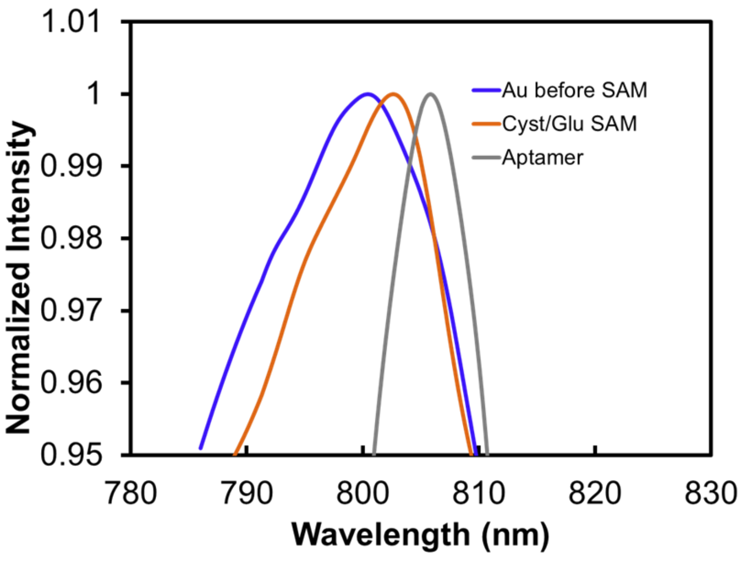

Figure S2. EOT due to SAM formations and aptamer immobilization.

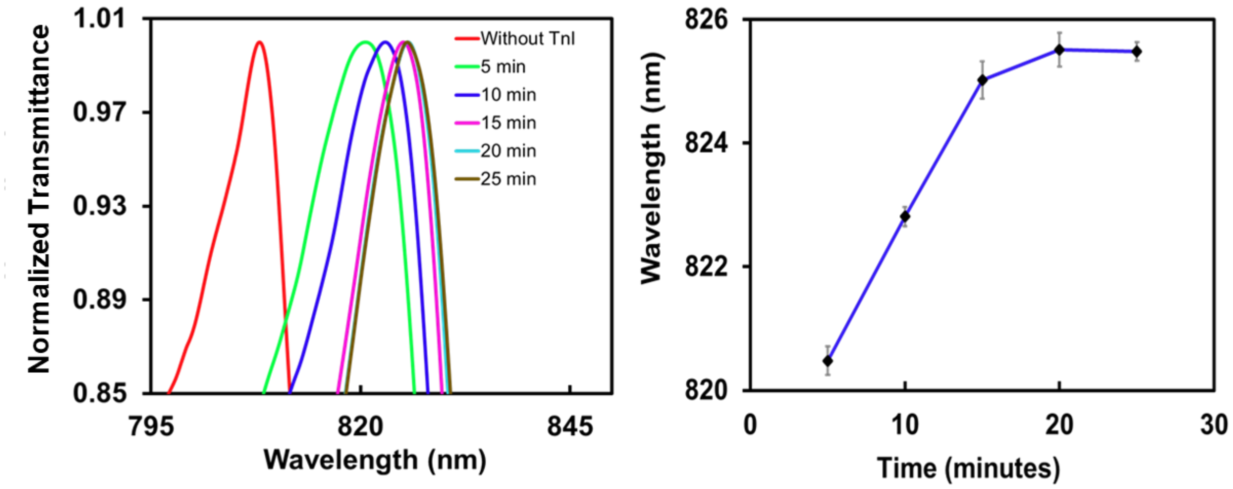

Figure S3. EOT upon TnI incubation with aptasensor at different binding times. This was obtained by transferring **50 µL** of 0.156 ng/mL cTnI sample in PBS on to a clean aptamer-modified sensor chip.

The Binding Event Analysis. The red shift in resonance EOT wavelength resulting from the increase in cTnI concentration **(Figure 2)** is taken to be due to the formation of a thin organic layer bound to the metal's surface. The thickness of the monolayer at different stages of the multi-layer formation is estimated based on Eq. 2.[8]

$$\Delta\lambda = m(n_{adlayer} - n_{air})[exp(-2d_1/\mathrm{l_d}][1 - exp(-2d_2/l_d)] \qquad (1)$$

The change in SPR response, $\Delta\lambda$, is defined as the shift in wavelength of the resonance peak in transmitted light intensity associated with changes in RI of material due to adlayer formation at the metal surface, and m is the sensitivity of the device measured in resonance wavelength shift per RIU obtained from the slop of the calibration curve shown in Figure 2B, The $n_{adlayer}$ (cystamine=1.49, glutaraldehyde=1.42) are the RIs of the adsorbed molecular layer, and that of the air, $n_{air} = 1$. The d_1 is the thickness of first or intermediate layer and d_2 represent the thickness of the second or subsequent adsorbed layer. The l_d is the SPR decay length. When the intermediate layer (for example cystamine, sandwiched between Au surface and glutaraldehyde) is very thin relative to l_d of the sensor, the dependence of l_d on RI approaches zero.[8] This mean that the influence of the intermediate layer on the responsivity of the sensor to the next added layer is negligible. Under such conditions, the parameter $exp(-\frac{2d_2}{l_d})$ in equation (1) approaches one. Hence, by rearranging Eq. 1, the relationship between $\Delta\lambda$ and adsorbed thickness, d, becomes.[8]

$$d = (l_d/2)\{\frac{\Delta\lambda}{[(m(n_{adlayer} - n_{air})]}\} \qquad (2)$$

To obtain the value of l_d, Eq. 3 was used to fit the experimental data as shown in **Figure S4**. The best fit was obtained using l_d=123 nm. Hence, the adlayer thickness due to surface functionalization and cTnI binding at different concentrations were estimated.

To determine the successful realization of thin-film layer formation arising from binding events at the metal's surface, the functionalization step, aptamer immobilization and cTnI binding was monitored by observing the wavelength (λ) peak shift during each step. The calculated effective thicknesses starting from the formation of SAM to the sensing of TnI at different concentrations is determined according to the measured average $\Delta\lambda$ as shown in **Table S1**. The average thickness of the cystamine/glutaraldehyde conjugate layer is ~1.10 nm using l_d=123 nm and m=384.0 nm/RIU, while the estimated aptamer thickness is ~1.15 nm using the DNA RI n_{DNA}=1.48.[9] Similarly, we calculated the effective adlayer thickness of binding TnI at different concentration (**Figure S5**). The thickness increase responds linearly to the change in resonance wavelength shift, $\Delta\lambda$, and correlate with the change in concentration.

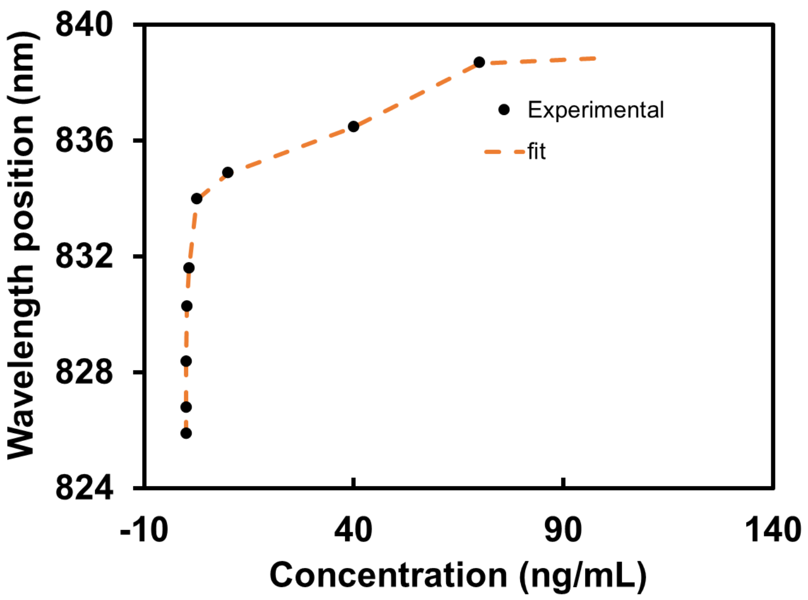

Figure S4. Graph of tSPR versus concentration (in PBS) showing experimental data points (black dots) fitted with the equation,

$$d = (l_d/2)\{\frac{\Delta\lambda}{[(m(n_{adlayer} - n_{air})]}\}$$

The best fit was obtained using l_d=123 nm.

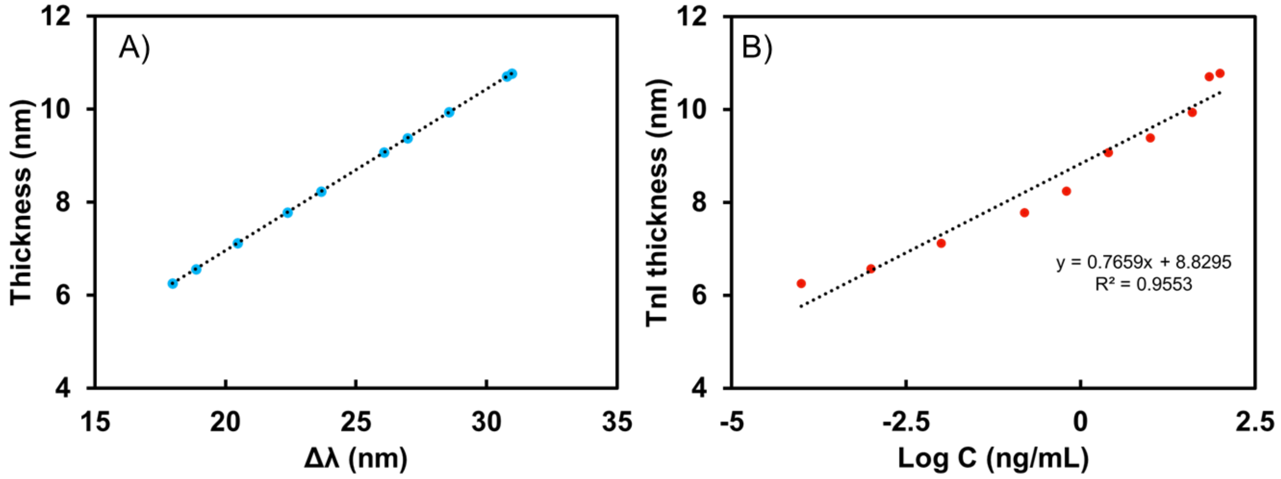

Figure S5: Dependence of adlayer film thickness on (A) change in wavelength position (B) cTnI concentration.

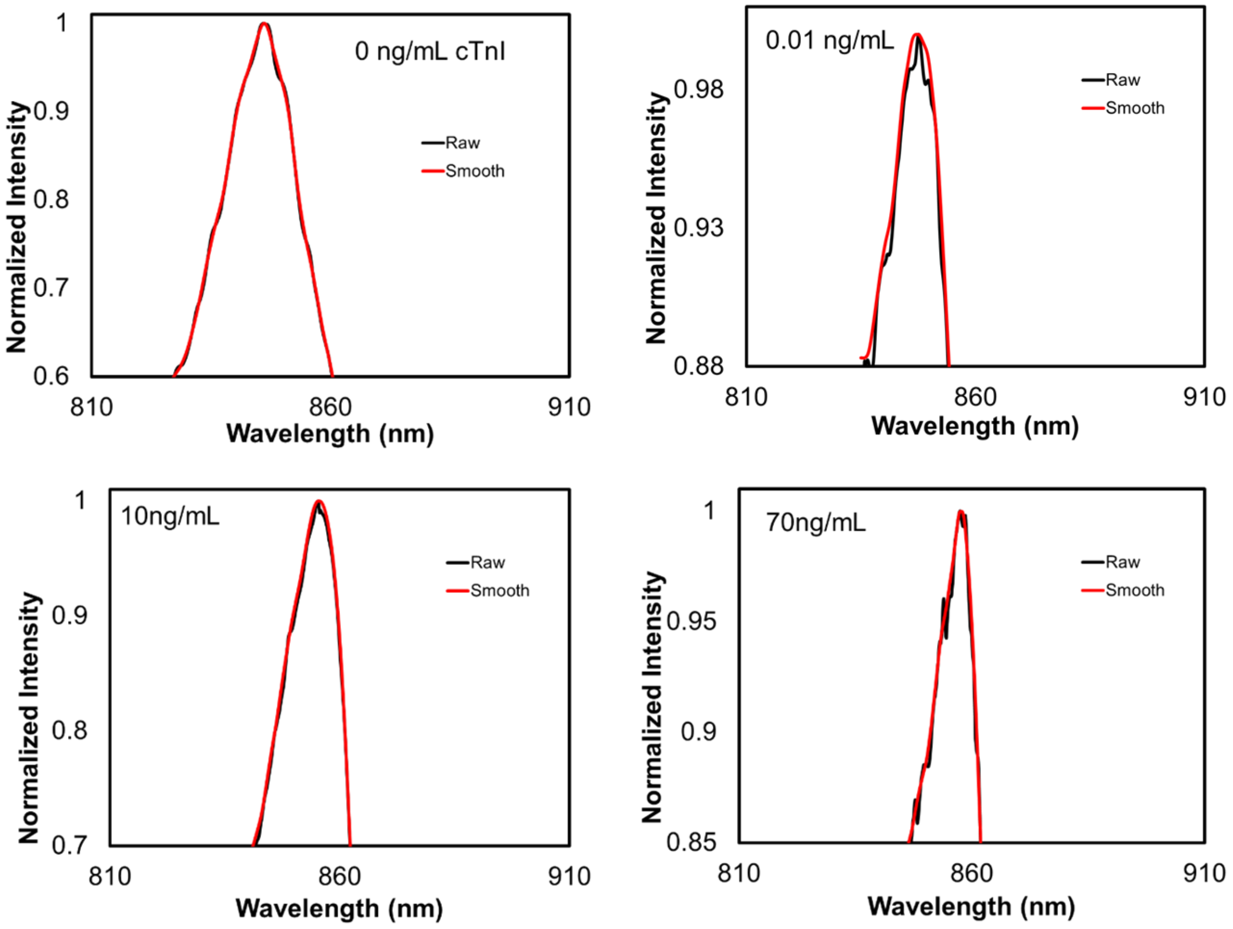

Figure S6. Representative plots of Raw and smoothed EOT spectrum curves.

A

B

C

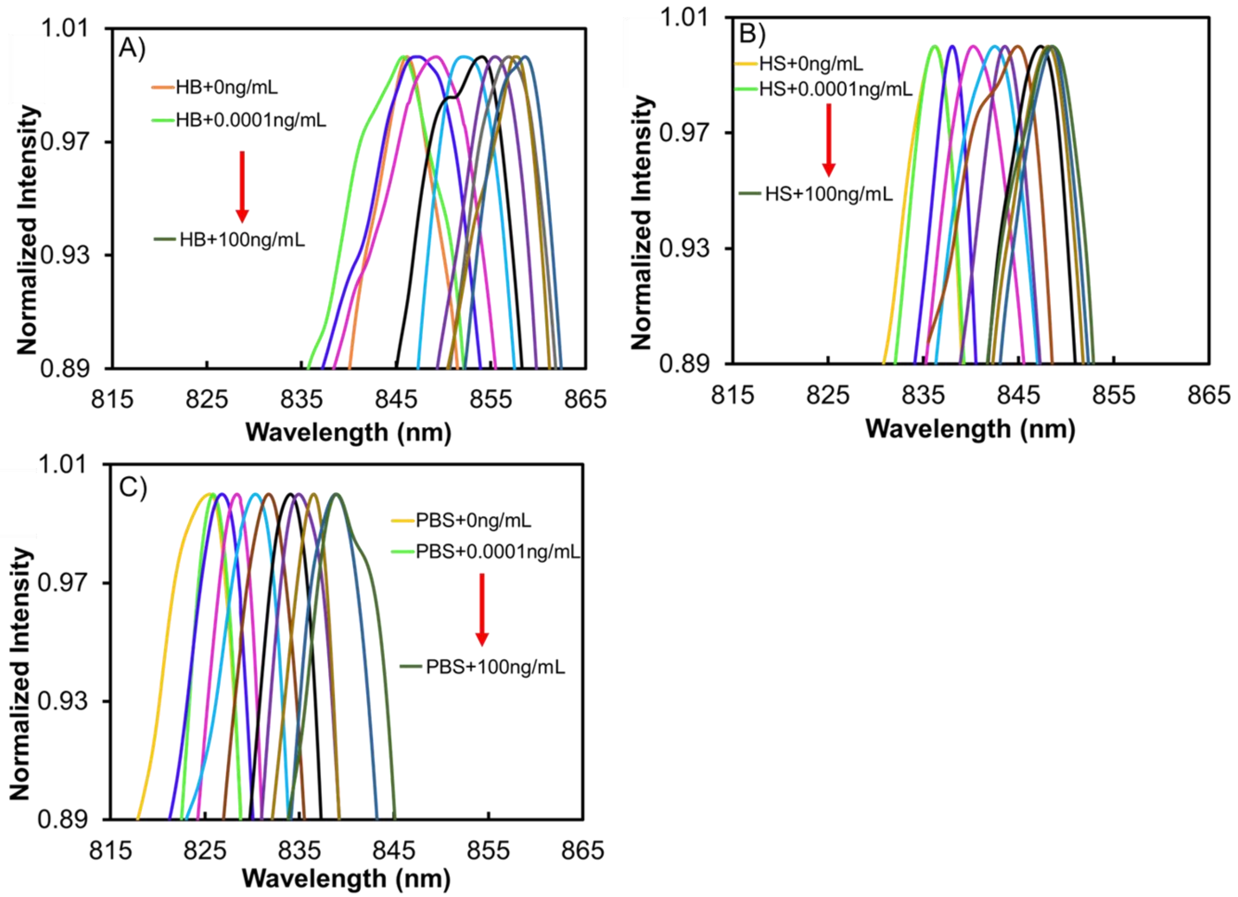

Figure S7. Enlarged EOT peak spectra for cTnI measurement in (A) Whole blood sample (B) Human serum (C) PBS buffer.

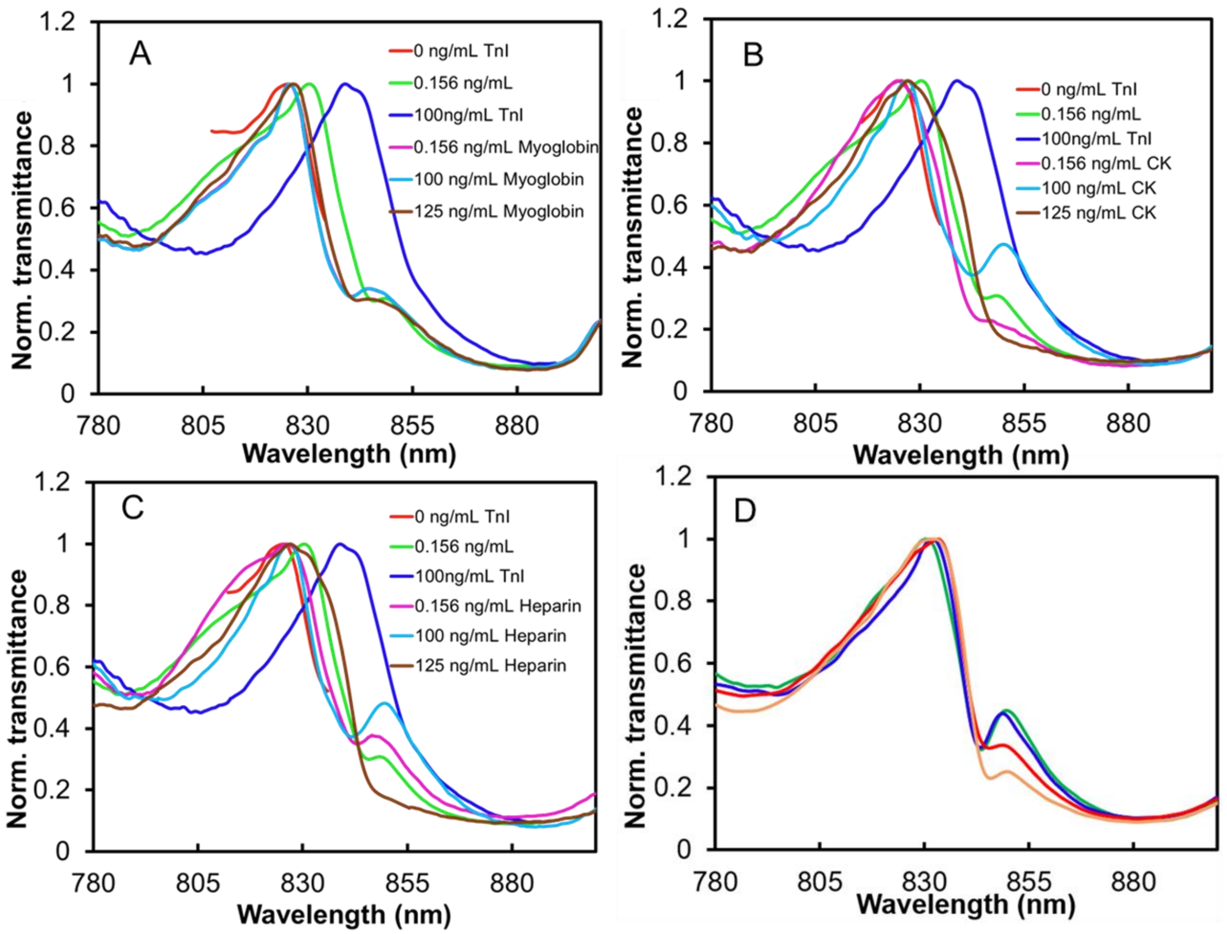

Figure S8. Normalized transmitted spectra of the NL sensor chip with (A) myoglobin, (B) heparin, and (C) creatin kinase in PBS, and (D) Transmission spectra recorded using 4 different fabricated aptasensor showing the reproducibility of the aptasensor at 2.5 ng/mL cTnI in PBS.

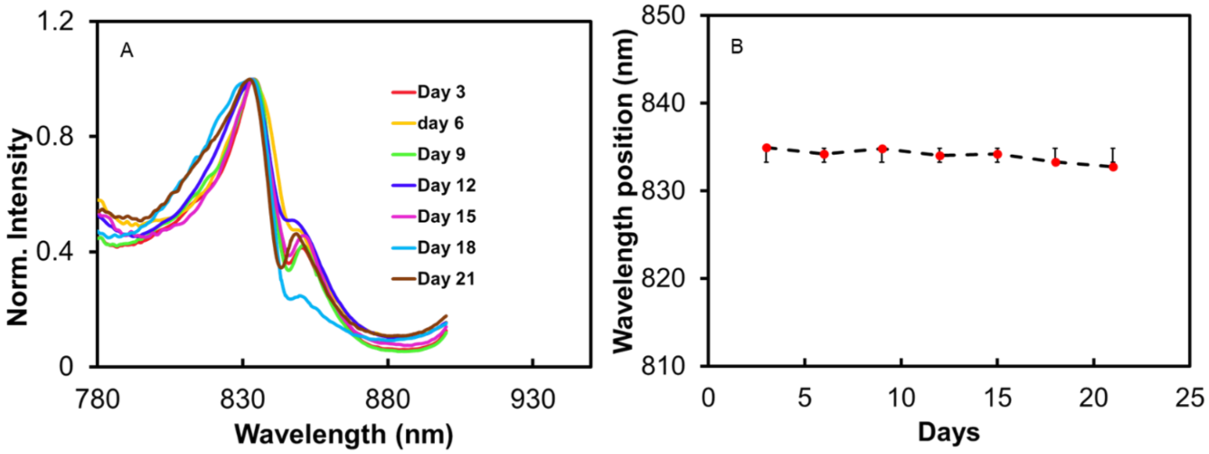

Figure S9. Stability of Aptasensor measured EOT spectra at 3 days interval for twenty-one days using 10 ng/mL TnI in PBS.

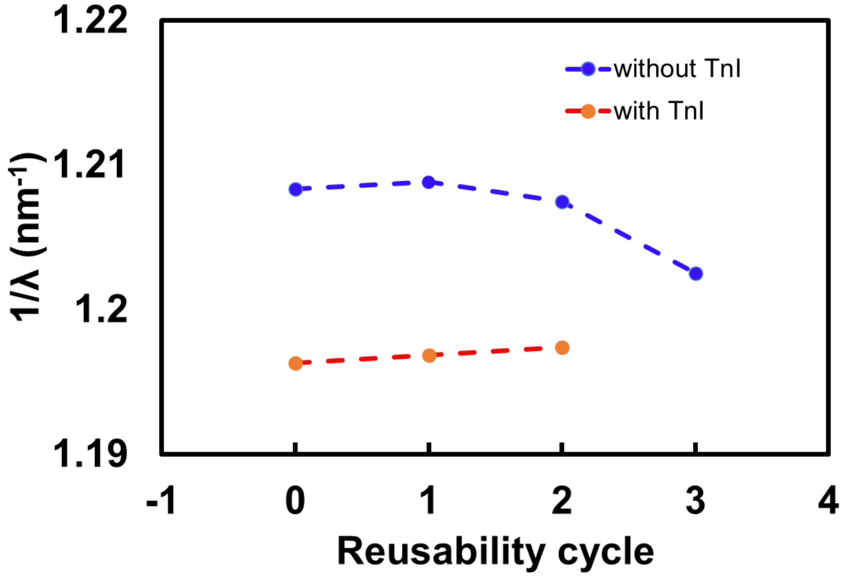

Figure S10. Reusability of aptasensor after 4 regeneration cycles using 10 ng/mL TnI in PBS

Table S1. Adlayer thickness as a function of concentration and wavelength peak shift (ld=123 nm)

	Cys	Glu	Apt	TnI concentration (ng/mL)									
				0.0001	0.001	0.01	0.156	0.625	2.5	10.0	40.0	70.0	100.0
Δλ (nm)	2.58± 0.64	1.37± 0.23	4.69± 0.08	17.97± 0.50	18.87 ±0.03	20.47 ±0.10	22.37 ±0.38	23.67 ±0.05	26.07± 0.11	26.97 ±0.02	28.57 ±0.63	30.77 ±0.03	30.97± 0.04
d (nm)	0.63	0.48	1.15	6.25	6.57	7.13	7.79	8.24	9.07	9.38	9.94	10.71	10.77

Reference

(1) Bethe, H. A. Theory of Diffraction by Small Holes. *Phys. Rev.* **1944**, *66*.

(2) Ebbesen, T. W.; Lezec, H. J.; Ghaemil, H. F.; Thiol, T.; Wolff, P. A. Extraordinary Optical Transmission through Sub-Wavelength Hole Arrays. *Nature* **1998**, *391* (6668), 667–669.

(3) Martín-Moreno, L.; García-Vidal, F. J.; Lezec, H. J.; Pellerin, K. M.; Thio, T.; Pendry, J. B.; Ebbesen, T. W. Theory of Extraordinary Optical Transmission through Subwavelength Hole Arrays. *Phys. Rev. Lett.* **2001**, *86* (6), 1114–1117. https://doi.org/10.1103/PhysRevLett.86.1114.

(4) Lalanne, P.; Sauvan, C.; Hugonin, J. P.; Rodier, J. C.; Chavel, P. Perturbative Approach for Surface Plasmon Effects on Flat Interfaces Periodically Corrugated by Subwavelength Apertures. *Phys. Rev. B* **2003**, *68*, 125404. https://doi.org/10.1103/PhysRevB.68.125404.

(5) Du, B.; Yang, Y.; Zhang, Y.; Jia, P. Enhancement of Extraordinary Optical Transmission and Sensing Performance through Coupling between Metal Nanohole and Nanoparticle Arrays. *J. Phys. D. Appl. Phys.* **2019**, *52*.

(6) Zheng Zeng, Xiaojun Shi, Taylor Mabe, Shaun Christie, Grant Gilmore, Adam W. Smith, and J. W. Protein Trapping in Plasmonic Nanoslit and Nanoledge Cavities: The Behavior and Sensing. *Anal. Chem.* **2017**, *89* (10), 5221–5229. https://doi.org/10.1021/acs.analchem.6b04493.

(7) Bagra, B.; Mabe, T.; Tukur, F.; Wei, J. A Plasmonic Nanoledge Array Sensor for Detection of Anti-Insulin Antibodies of Type 1 Diabetes Biomarker. *Nanotechnology* **2020**, *31* (32). https://doi.org/10.1088/1361-6528/ab8c05.

(8) Jung, L. S.; Campbell, C. T.; Chinowsky, T. M.; Mar, M. N.; Yee, S. S. Quantitative Interpretation of the Response of Surface Plasmon Resonance Sensors to Adsorbed Films. *Langmuir* **1998**, *14* (19), 5636–5648. https://doi.org/10.1021/la971228b.

(9) Rajzler, V. A. P.; Ung, W. O. J.; Yunghwan, K. O. H.; Ajzl, J. A. C.; Ekvindova, P. A. N. Optical Properties of Deoxyribonucleic Acid Thin Layers Deposited on an Elastomer Substrate. *Opt. Mater. Express* **2020**, *10* (2), 421–433.
